# Two proteocephalid cestodes in the fish *Malapterurus electricus* and *Heterobranchus bidorsalis* from Lake Nasser, Egypt: a morphological, molecular, and histopathological study

**DOI:** 10.1186/s12917-024-04048-1

**Published:** 2024-05-20

**Authors:** Awatef Hamed Hamouda, Abuelhassan Elshazly Younis

**Affiliations:** 1 Fish Health and Diseases Department, Faculty of Fish and Fisheries Technology, Aswan University, Aswan, 81528 Egypt; 2 Zoology Department, Faculty of Science, Aswan University, Aswan, 81528 Egypt

**Keywords:** *Corallobothrium solidum*, *Heterobranchus bidorsalis*, Histopathology, *Malapterurus electricus*, Molecular identification, *Proteocephalus sp*.

## Abstract

Despite the importance of the electric catfish (*Malapterurus electricus*) and the African giant catfish (*Heterobranchus bidorsalis*) in the foodweb of Lake Nasser, Egypt, little is known about their diseases and parasitic fauna. This work describes, for the first time, cestodiasis in *M. electricus* and *H. bidorsalis*. *Corallobothrium solidum* and *Proteocephalus* sp. were identified morphologically and molecularly from *M. electricus* and *H. bidorsalis*, respectively. Using PCR, sequencing, and phylogenetic analysis, the two cestodes shared rRNA gene sequence similarities yet were unique and the two new sequences for the proteocephalid genera were submitted to the GenBank database. The prevalence of infection was 75% and 40% for the two fish species, respectively. Infections significantly increased in the summer and spring and were higher in female fish than in male fish. The intestine was the preferred site of the two adult cestodes. However, in the case of *C. solidum* some larval cestodes were found outside the intestine in between the skin and abdominal musculature, attached to the mesentery, and within intestinal tunica muscularis. Desquamation of the intestinal epithelium and inflammation at the site of infection in addition to congestion of the intestinal wall of the tapeworm infected fish were evident, indicating that *C. solidum* and *Proteocephalus* sp. impacted the infected fish. The larval stages of *C. solidum* attempted to penetrate the intestine and sometimes they were encircled within fibrous layers infiltrated with inflammatory cells. The infected fish’s musculature was free of cestode infections. Preventive measures should be implemented to prevent the spread of infections.

## Introduction

There are approximately 461 species of cestodes that parasitise teleosts out of the known 4,810 species of cestodes that exist in the world [1]. Cestodes exhibit a high degree of host specificity and are common in all of Africa’s major water systems [2]. These are extremely effective parasitic infections in fish [3]. They mostly affect the gastrointestinal tract, particularly the intestine of vertebrates, and their larvae can be found in a variety of invertebrate and vertebrate body cavities as well as other organs [4]. Certain parasitic cestode infections harming fish are seen as an economic issue; they have an impact on fish weight gain, decreasing marketability, and occasionally, in severe cases, increasing mortality rates, leading to increased financial losses [5–7].

Proteocephalidea, which has six genera and nineteen species, is one of the most significant orders of cestodes [8]. Within the family Proteocephalidae, the genus *Proteocephalus* is a globally distributed parasitizing amphibians, reptiles, and fish [9, 10]. All Proteoc*ephalus* species have planktonic crustaceans, diaptomids, or cyclopid copepods as intermediate hosts, and vertebrates as final hosts, contracting the infection by ingesting them [11].

*M. electricus* and *H. bidorsalis* are among fish species found in Lake Nasser [12, 13]. Within the Malapteruridae family of electric catfishes, *M. electricus* is one of the most significant species currently known to exist in Africa [14, 15]. Its name comes from the electric organ that produces 300–400 Vs and forms a covering beneath the skin that surrounds the body. This covering is utilized as a defense mechanism and to catch prey [16]. Fresh or smoked *M. electricus* is highly prized by customers in Africa [17].

Furthermore, the *Heterobranchus* genus, which includes four significant species, including *H. bidorsalis*, is one of the most economically significant genera in the family Clariidae [14]. The African giant catfish, *H. bidorsalis*, is a very commercial species in African countries that performs better than other species in the family Clariidae [18]. With its ability to grow up to 1.2 m in length and 30 kg in weight, along with its ability to mature quickly in captivity (10–12 months), and it’s delicious and high-quality meat, this fish has a great deal of potential for aquaculture, particularly in Africa [19, 20].

The parasitic diseases that infect the fish in Lake Nasser are generally poorly understood [21–23]. This is especially true for the diseases of the two ecologically important fish species *M. electricus* and *H. bidorsalis* [24]. In order to better understand the cestodes causing the two types of catfish infections, this study will employ morphological, molecular, and phylogenetic analysis techniques to examine the cestodes. Clinical signs, postmortem lesions, organ or tissue susceptibility, prevalence, seasonal incidence, intensity, and histopathological changes will also be recorded.

## Materials and methods

### Study area

**Fig. 1:**
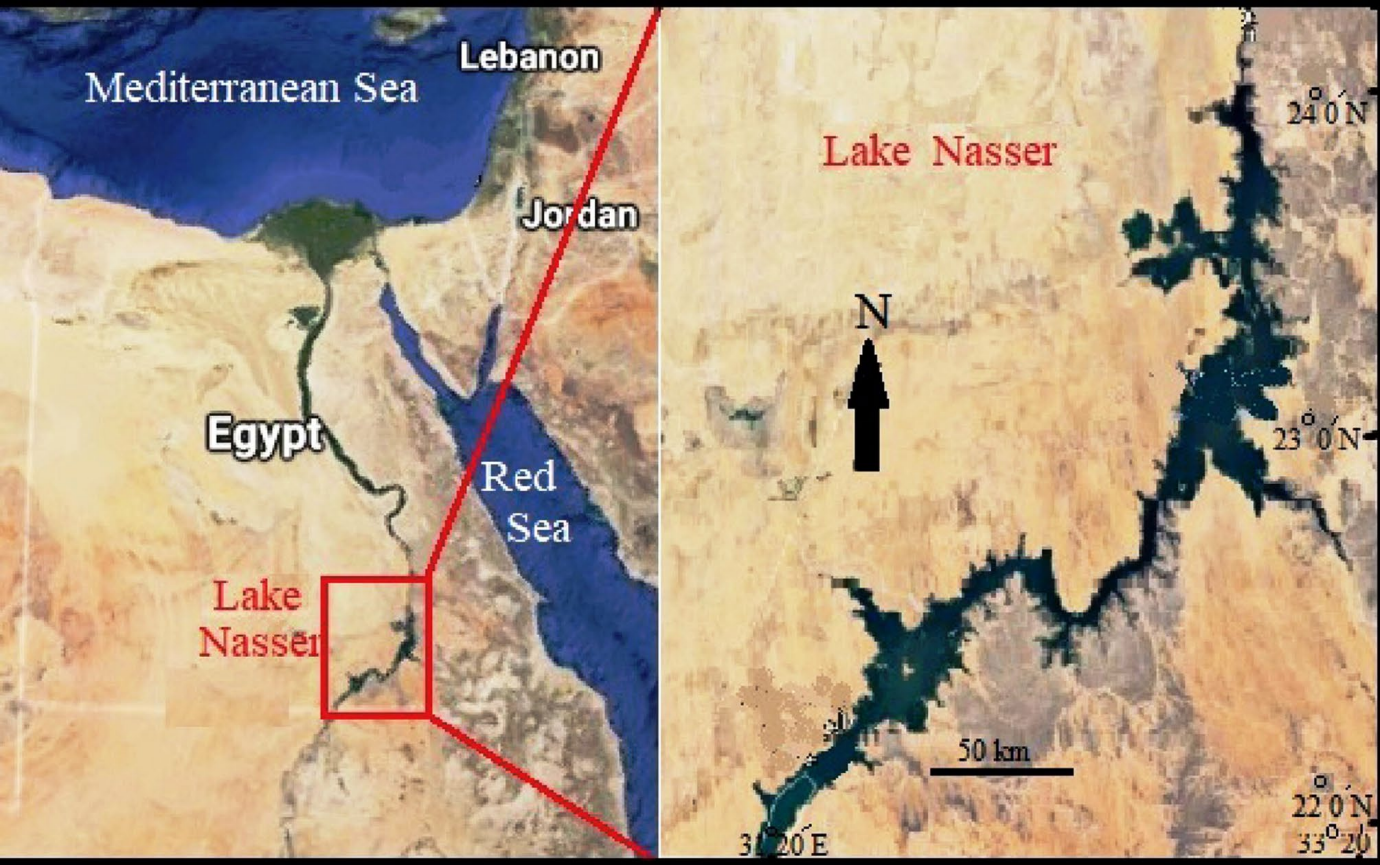
Location map of Lake Nasser, Aswan Governorate, Egypt

This study was conducted on two catfishes; *M. electricus* and *H. bidorsalis* from Lake Nasser. The lake is located in southern Egypt and is also known as the High Dam Lake. It was created after the construction of the High Dam across the Nile in Aswan between 1960 and 1970 (Fig. 1). The lake supports the fishing industry in Egypt and extends over 500 km; 350 km in Egypt and 150 km in Sudan. It has an average width of approximately 12 km at a water level of 180 m [25].

### Fish

During the period from May 2020 to April 2021, 300 fish specimens—200 *M. electricus* (47.5 ± 0.3 cm x 1650 ± 0.42 g) and 100 *H. bidorsalis* (60 ± 0.15 cm x 1628 ± 0.34 g)—were bought live from several fishermen on Lake Nasser. For each season, a quarter of the total fish were bought. The fish were transported to the Laboratory of Fish Health and Diseases at Aswan University’s Faculty of Fish and Fisheries Technology for parasitological examination.

### Clinical signs and postmortem examination

Upon arriving the laboratory, the fish were euthanized a head blow immediately, followed by cervical dislocation according to the methods detailed by AVMA [26]. After that, total length of the fish measured (cm) and weighed (g), and their sex was determined as recorded by Eissa [27]. The external and internal gross lesions were recorded as defined by Eissa [27]. The infected fish were photographed.

### Parasitological examination

Cestodes were observed with the naked eye or magnifying glass due to their large size, whitish color, or movements and collected. All internal organs, especially the intestine, intestinal wall, gall bladder, peritoneal cavity, and musculature, were examined to detect the cestodes or their detached proglottids. The recovered cestodes were cleaned and thoroughly rinsed in a saline solution to remove debris. They were then divided into two parts: some were preserved in alcohol-formalin-acetic acid until staining time, and some were kept in 70% ethyl alcohol at − 20 °C for molecular study. The preserved cestodes were stained with acetic acid alum carmine, dehydrated with ascending concentrations of ethyl alcohol, cleared in xylene, and finally mounted in Canada balsam according to Hamouda [6]. An Olympus DP74 camera attached to an Olympus BX43 microscope was used to examine and photograph the stained cestodes. Squash preparations of the musculature and internal organs were done by compressing small pieces of them between two glass slides and examining them under a stereomicroscope for the detection of cestode larvae. The number of recovered cestodes was counted per fish. The prevalence, mean intensity, and mean abundance of cestodal infections were recorded according to Bush et al. [28].

### Identification of collected cestodes

*C. solidum* and *Proteocephalus* sp. were identified using taxonomic keys by Rego [9], Ibraheem [29], and Scholz et al. [30].

### Molecular identification and phylogenetic analyses

Genomic DNA (gDNA) was extracted from each individual ethanol-preserved tapeworm using a phenol/chloroform technique as described by Younis et al. [31]. The parasite materials were, in short, precipitated with 5.2 M ammonium acetate after being digested with proteinase K in ALT buffer for a whole night at 56 °C (Dneasy Kit, Qiagen). The samples were diluted with 20–50 µL of ddH2O based on the size of the pellets. A Nanodrop spectrophotometer (Implen NP80, Germany) was used to determine the gDNA sample concentrations spectrophotometrically. Forward F2 (GTCGTAACAAGGTTTCCGTAGGTG) and reverse R2 (TATGCTTAAGTTCAGCGGGTAATC) primers were utilized to amplify the whole ITS region according to Brabec et al. [32]. A total of 50 µL of 1× thermal buffer (MyTaq Red Reaction buffer, Bioline, UK), 10–20 pmol forward and reverse primers, 10 mM dNTPs mix (Alliance Bio, USA), 5 u/µL Taq polymerase (Bioline, UK), and 0.1–0.2 µg gDNA were used in the PCR amplifications. In a thermocycler (Sensoquest Lab cycler, SensoQuest GmbH, Germany), the amplifications were carried out under the following cycling conditions: 5 min of initial denaturation at 95 °C, 30 s of 94 °C, 30 s of annealing at 55 °C, 30 s of elongation at 72 °C, and 2 min of final extension at 72 °C. As controls, samples with fish intestinal DNA or without gDNA were added to the PCR. On a 1% agarose gel, amplification products were examined and stained with ethidium bromide. Next, using ultraviolet (UV) gel documentation equipment (UVP Bio-Doc IT-220 Imaging System, BioExpress, USA), the DNA bands were immediately observed and photographed. The PCR products were purified using the DNA Clean and Concentrator Kit (Zymo Research, USA) in accordance with the manufacturer’s procedure. Using the same primers as in the original PCR, the refined products of PCR were sequenced in each direction (forward and reverse). An automated DNA sequencing machine (Model 3730XL) from Applied BioSystems (USA) and the dideoxy termination method (Macrogen Inc., Korea) were used. For each sequence, homology searches were performed using the NCBI Blast software (ncbi.nlm.nih.gov). After that, each forward sequence was manually altered and put together using the CAP3 algorithm after being compared to its reverse counterpart. Then, using the multiple sequence alignment tool CLUSTALW, the produced sequences were aligned to the most homologous sequences in the database as well as to each other. Phylogeny.fr, an online application, was used to construct phylogenetic trees. MUSCLE (v3.8.31) was used for alignment, and its default parameters were optimized for maximum accuracy. Gblocks (v0.91b) were used to eliminate unclear sections (those with gaps and/or misalignment) following alignment. The maximum likelihood technique (v3.1/3.0 aLRT) of the PhyML program was then used to reconstruct the phylogenetic tree. Lastly, the phylogenetic tree was edited and graphically represented using TreeDyn (v198.3) according to Dereeper et al. [33].

### Histopathological examination

Parts of the infected intestines with *C. solidum* and *Proteocephalus* sp. of *M. electricus* and *H. bidorsalis* were preserved in 10% neutral buffered formalin. The samples were dehydrated in ascending concentrations of ethyl alcohol, embedded in paraffin wax, and then sectioned using a microtome to a thickness of 5 μm according to Suvarna et al. [34]. The sectioned samples were stained with hematoxylin and eosin, as described by Bancroft and Gamble [35].

### Statistical analysis

The seasonal prevalence data were analyzed using one-way ANOVA tests followed by Duncan-test as a post-hoc (SPSS version 22, SPSS Inc., Il, USA). The level of significance was accepted at *P* < 0.05 According to Greenland et al. [36]. All data are presented as means ± standard error (SE).

## Results

### Clinical signs and postmortem examination

*M. electricus* and *H. bidorsalis* infected with *C. solidum* and *Proteocephalus* sp., respectively, exhibited no gross abnormalities, except that the heavily infected fish were emaciated and had distended abdomens.

**Fig. 2:**
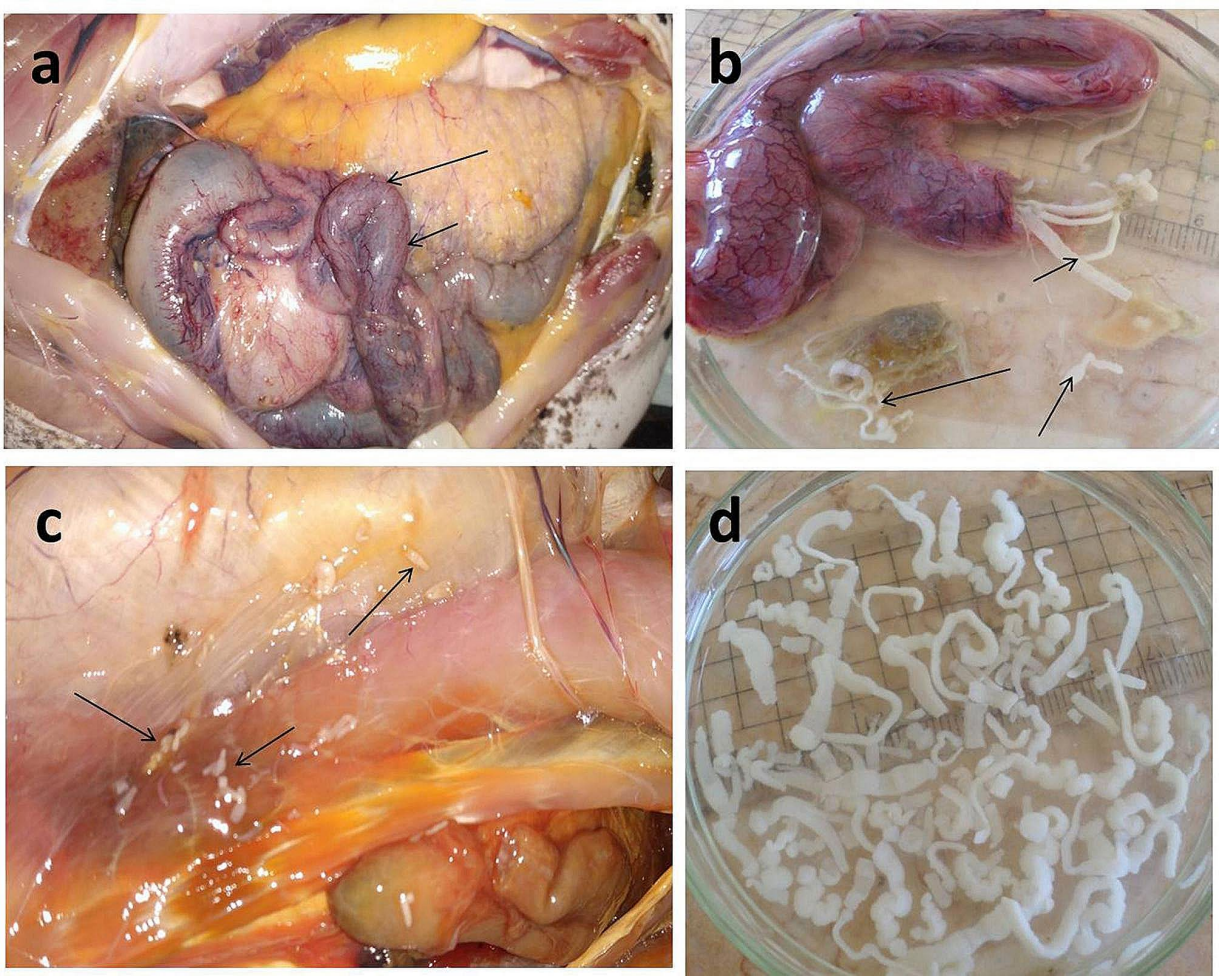
*Malapterurus electricus *infected with *Corallobothrium solidum.***a**: Highly congested intestine (arrows) infected with large number of *Corallobothrium solidum*. **b**: Large number of *Corallobothrium solidum* (arrows) liberating from the congested intestine. **c**: *Corallobothrium solidum* (arrows) between the skin and abdominal musculature. **d**: Large number of *Corallobothrium solidum* recovered from the intestine of *Malapterurus electricus*

**Fig. 3:**
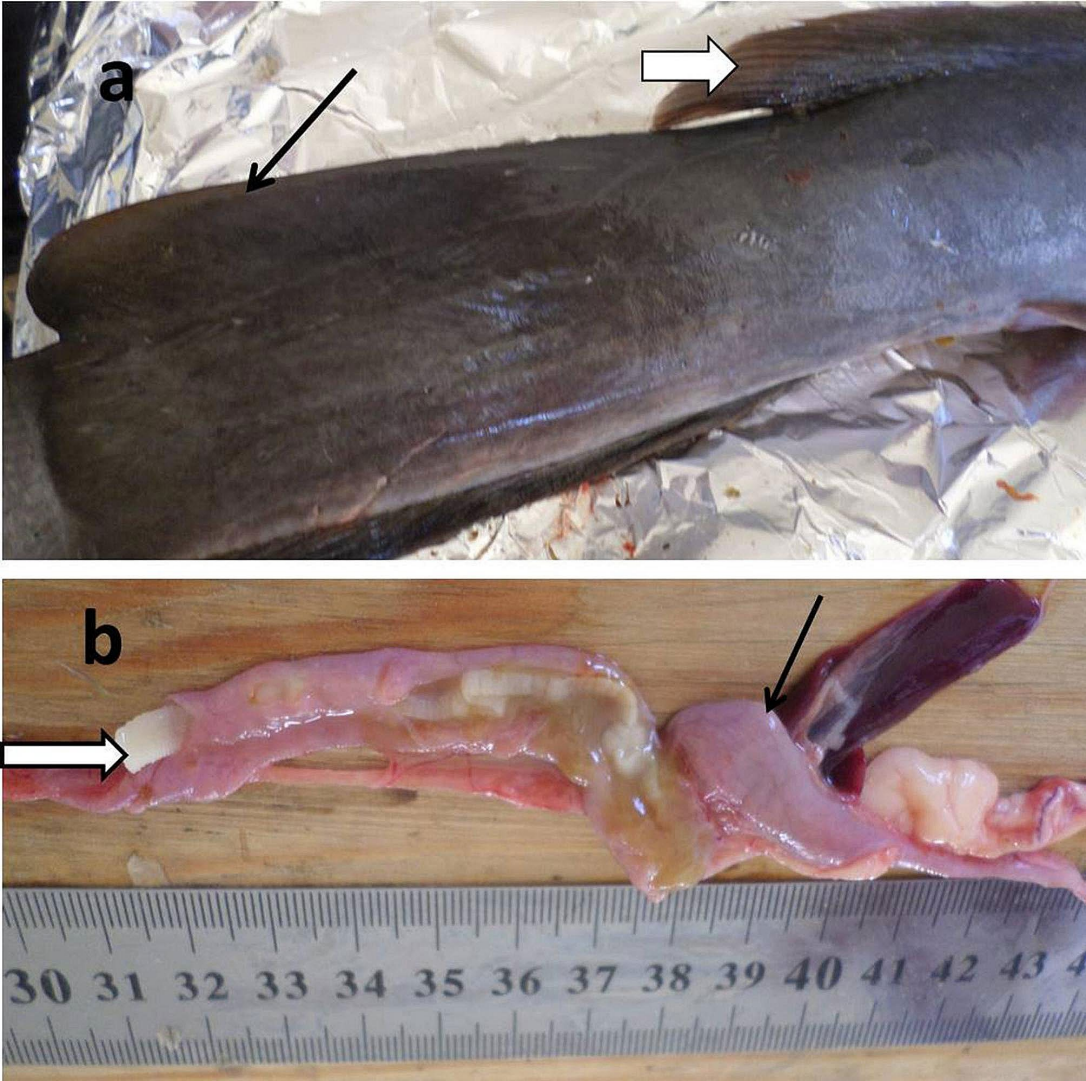
*Heterobranchus bidorsalis*. **a**: Adipose fin (black arrow) and dorsal fin (black bordered white arrow). **b**: Congested intestine (black arrow) infected with *Proteocephalus* sp. (black bordered white arrow) with increased mucus secretion

The internal organs of infected *M. electricus* and *H. bidorsalis* were highly congested, particularly the infected intestines, Fig. (2) a, b, & Fig. (3) b, with increased mucus (3b).

*M. electricus* was infected with two forms of *C. solidum* (adult and larval stages) and to our knowledge; it is the first time to detect adult and larval stages of a particular cestode in the same host. Adult *C. solidum* was attached to the middle portion of the intestine but its larval stages were found outside the intestine in between the skin and abdominal musculature, Fig. (2) c, and attached to the mesentery of infected fish. While in *H. bidorsalis*, it was infected with adult *Proteocephalus* sp. that was exclusively attached to the middle portion of the intestine. Interestingly, there were no cestodes, either adult or larvae, in the musculature of the infected *M. electricus* and *H. bidorsalis*.

Recovered adult *C. solidum* and *Proteocephalus* sp. were white in color, reach up to 10 cm in length, highly active, and attained remarkable scoleces, Fig. (2) d & Fig. (3) b.

*H. bidorsalis* is characterized by the presence of a longer dorsal fin compared to its adipose fin and the absence of a black spot at its tail end, Fig. (3) a.

### Parasitological examination

*C. solidum* and *Proteocephalus* sp. were prevalent all year in Lake Nasser; *C. solidum* was recorded in *M. electricus*, with a prevalence of 75% and infection intensities ranging from 1 to 50 cestodes per fish. Meanwhile, *Proteocephalus* sp. was recorded in *H. bidorsalis* with a prevalence of 40% and infection intensities ranging from 1 to 4 cestodes per fish. Table 1 showed the prevalence, intensity, and mean abundance of infections with *C. solidum* and *Proteocephalus* sp. in *M. electricus* and *H. bidorsalis*, respectively, from Lake Nasser in the period from May 2020 to April 2021.

**Table 1:** Prevalence, mean intensity and mean abundance of *Corallobothrium solidum* and *Proteocephalus* sp. in *Malapterurus electricus* and *Heterobranchus bidorsalis* respectively from Lake Nasser in the period from May 2020 to April 2021

Fish species	No. exam. fish	No. infected fish	Prevalence %	No. parasites	Mean intensity	Range of intensity	Mean abundance
***Malapterurus electricus***	200	150	75	1650	11	1–50	8.25
***Heterobranchus bidorsalis***	100	40	40	85	2.125	1–4	0.85

The female fish recorded higher infection rates than the male ones, as shown in Table 2.

**Table 2:** Rate of infections in male and female *Malapterurus electricus* and *Heterobranchus bidorsalis* infeted with *Corallobothrium solidum* and *Proteocephalus* sp. respectively from Lake Nasser in the period from May 2020 to April 2021

Fish species	Total No. of examined fish		No. of infected fish		% of infection	
*Malapterurus* *electricus*	200		150		75	
	Male	Female	Male	Female	Male	Female
	140	60	102	48	72.85	80
*Heterobranchus bidorsalis*	100		40		40	
	Male	Female	Male	Female	Male	Female
	75	25	29	11	38.67	44

**Fig. 4:**
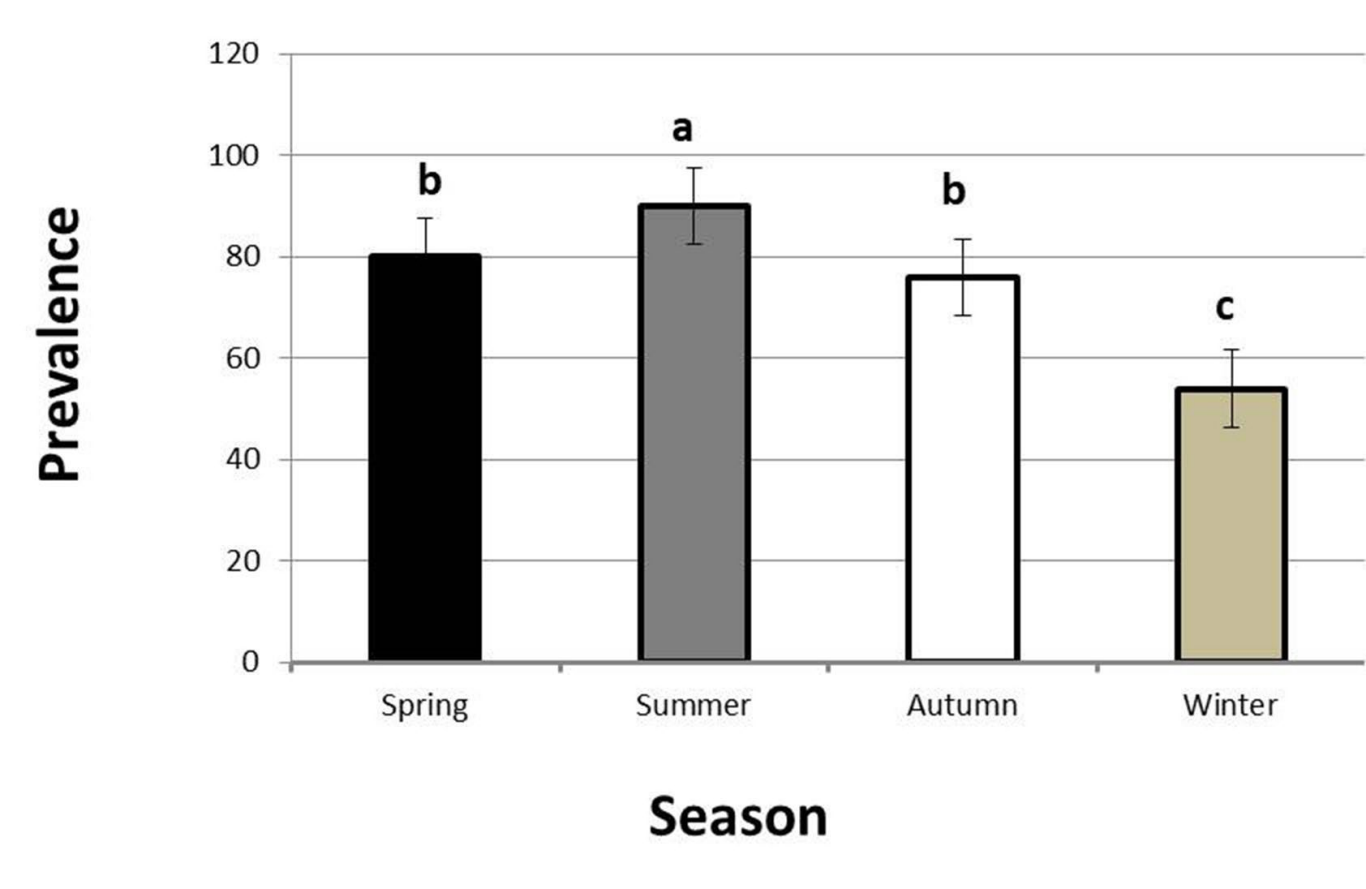
Seasonal prevalence of *Corallobothrium solidum* in *Malapterurus electricus* from Lake Nasser in the period from May 2020 to April 2021. Bars with different letters are significantly different (*P* < 0.05). Values are expressed as mean ± SE. *n* = 200

**Fig. 5:**
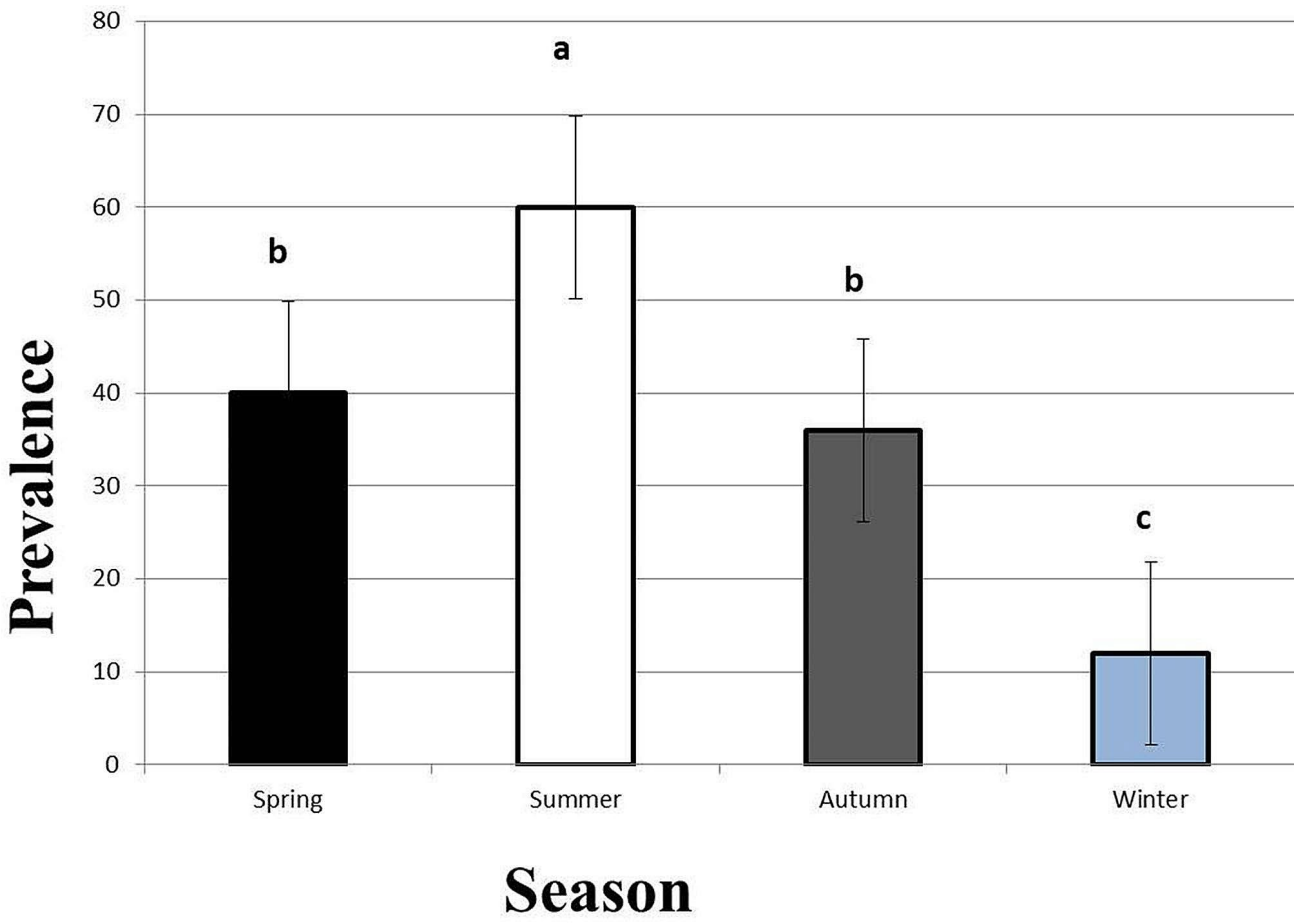
Seasonal prevalence of *Proteocephalus* sp. in *Heterobranchus bidorsalis* from Lake Nasser in the period from May 2020 to April 2021. Bars with different letters are significantly different (*P* < 0.05). Values are expressed as mean ± SE. *n* = 100

*C. solidum* and *Proteocephalus* sp. recorded higher infection rates in the summer (90%, 60%), spring (80%, 40%), autumn (76%, 36%), and winter (54%, 12%) in a descending manner, as shown in Figs. 4 and 5. The recorded prevalence showed significant variations between summer and winter and between them and the other seasons (*P* < 0.05), while spring and autumn showed insignificant variations (*P* > 0.05).

### Identification of collected cestodes

Only two cestodes were identified in this study from *M. electricus* and *H. bidorsalis*. Based on their morphological criteria, these two cestodes were *C. solidum* and *Proteocephalus* sp. respectively that belonged to Proteocephalidae because the testes, ovary, vitelline follicles, and uterus were medullary in position.

**Fig. 6:**
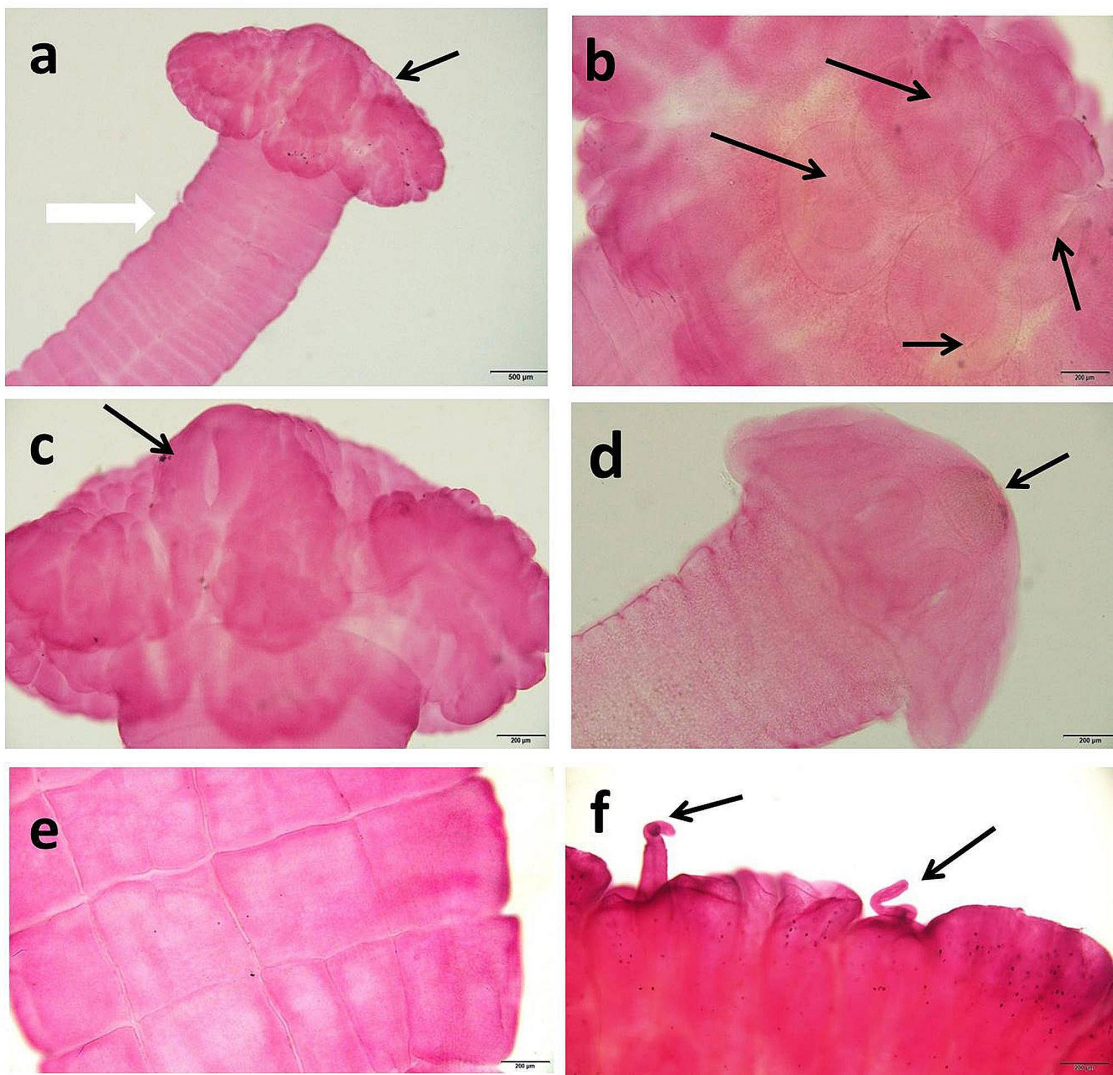
*Corallobothrium solidum* isolated from *Malapterurus electricus* stained with acetic acid alum carmine. **a**: Scolex (black arrow) and strobila (white arrow), bar = 500 μm. **b**: Four suckers (arrows) in the scolex, bar = 200 μm. **c**: U- shape sucker (arrow) in the scolex, bar = 200 μm. **d**: Larval developmental stage showing apical organ (arrow), bar = 200 μm. **e**: Longitudinal and transverse grooves on the strobili, bar = 200 μm. **f**: Cirrus everted (arrows) from the strobili, bar = 200 μm

The cestode recovered from *M. electricus* was identified as *C. solidum*. Live *C. solidum* was white in color and reached up to ten centimeters long, Fig. (2) d. Scolex was large, umbrella shape with well-developed metascolex, much wider than the proliferative zone (neck). Metascolex was well-developed, formed a folded collar surrounding suckers. Suckers were large, uniloculate, and deeply embedded; the external (outer) margins of suckers had semispherical (U-shaped, i.e., interrupted anteriorly) musculature serving as a sphincter, Fig. (6) a, b, c. The larval cestode had a large apical organ that was spherical to oval in shape, and the metascolex was not fully developed, Fig. (6) d. Body surface with deep longitudinal and transverse grooves (wrinkles) forming a rectangular network (internal longitudinal musculature weakly developed, formed by a narrow band of isolated muscle fibers, more dense on the lateral sides of the strobili), Fig. (6) e. Testes were medullary, spherical to oval, in 2 irregular (incomplete) layers, in 2 lateral fields connected anteriorly. Cirrus-sac was oval and may be everted, Fig. (6) f. The ovary was medullary, compact, with small follicles on the surface, bilobed, and had short and wide lateral lobes connected by a ventrally situated isthmus. Vitelline follicles were medullary, small, and arranged in two narrow lateral bands. The uterus was a medullary uterine stem with numerous intensely staining cells concentrated along its wall in immature proglottids.

**Fig. 7:**
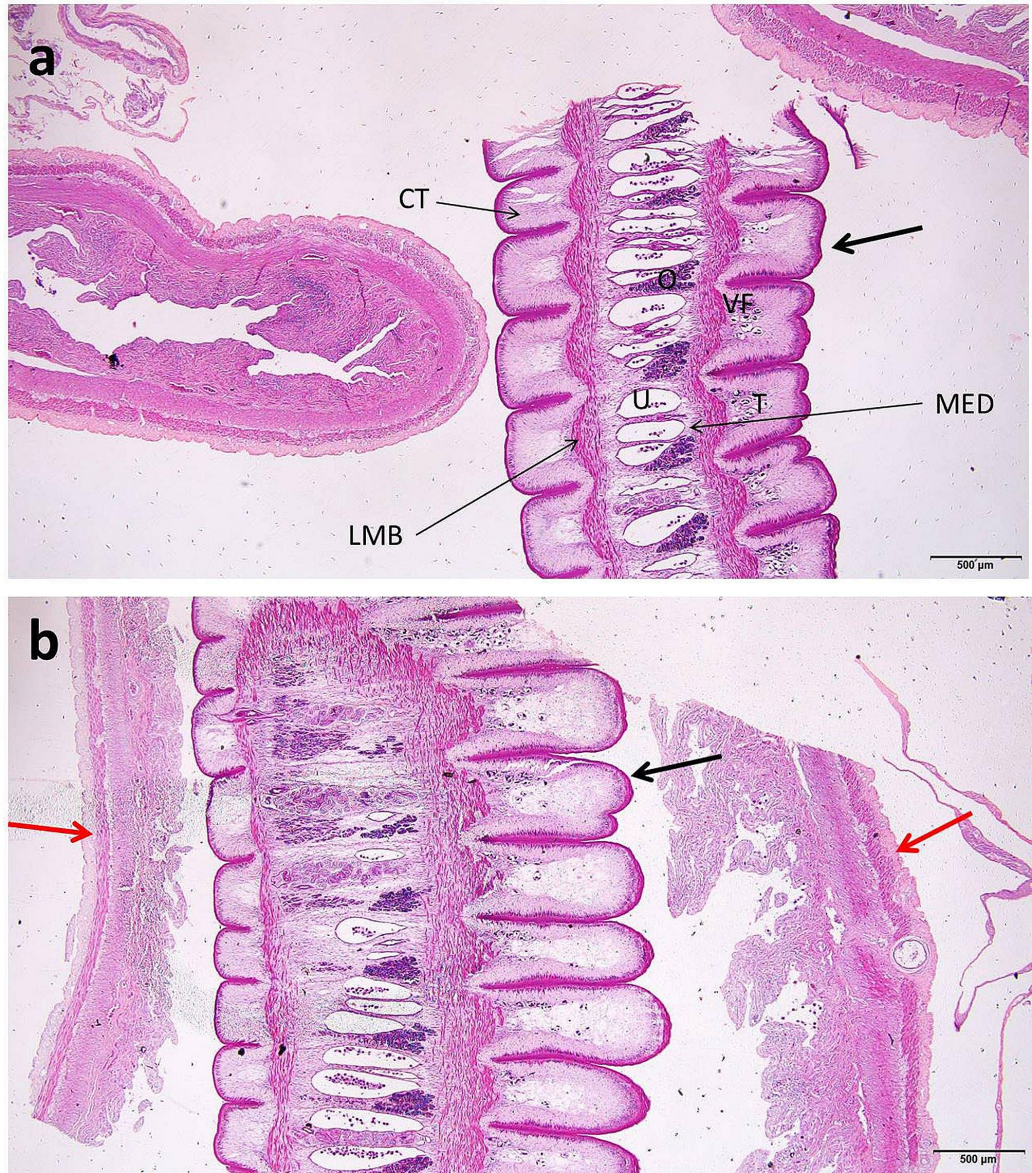
*Heterobranchus bidorsalis*^,^s intestine. **a**: *Proteocephalus* sp. in the lumen (arrow), H&E, bar = 500 μm. Abbreviations: CT, cortex; LMB, longitudinal muscle bundle; MED, medulla; O, ovary; T, testes; U, uterus; VF, vitelline follicles. **b**. *Proteocephalus* sp. (black arrow) nearly blocks the intestinal lumen, and the red arrows indicate the intestinal wall, H&E, bar = 500 μm

The cestode recovered from *H. bidorsalis* was identified as *Proteocephalus* sp.; live cestode was white in color and reach up to ten centimeters long, Fig. (3) b. The carmine-stained specimens do not show any characteristics because the cestode was highly contracted and very thick; only four suckers can be seen on the anterior end of the cestode. By histopathogical examinations, the testes, ovary, vitelline follicles, and uterus were seen medullary in position, Fig. (7) a, b.

### Molecular identification and phylogenetic analyses

**Fig. 8:**
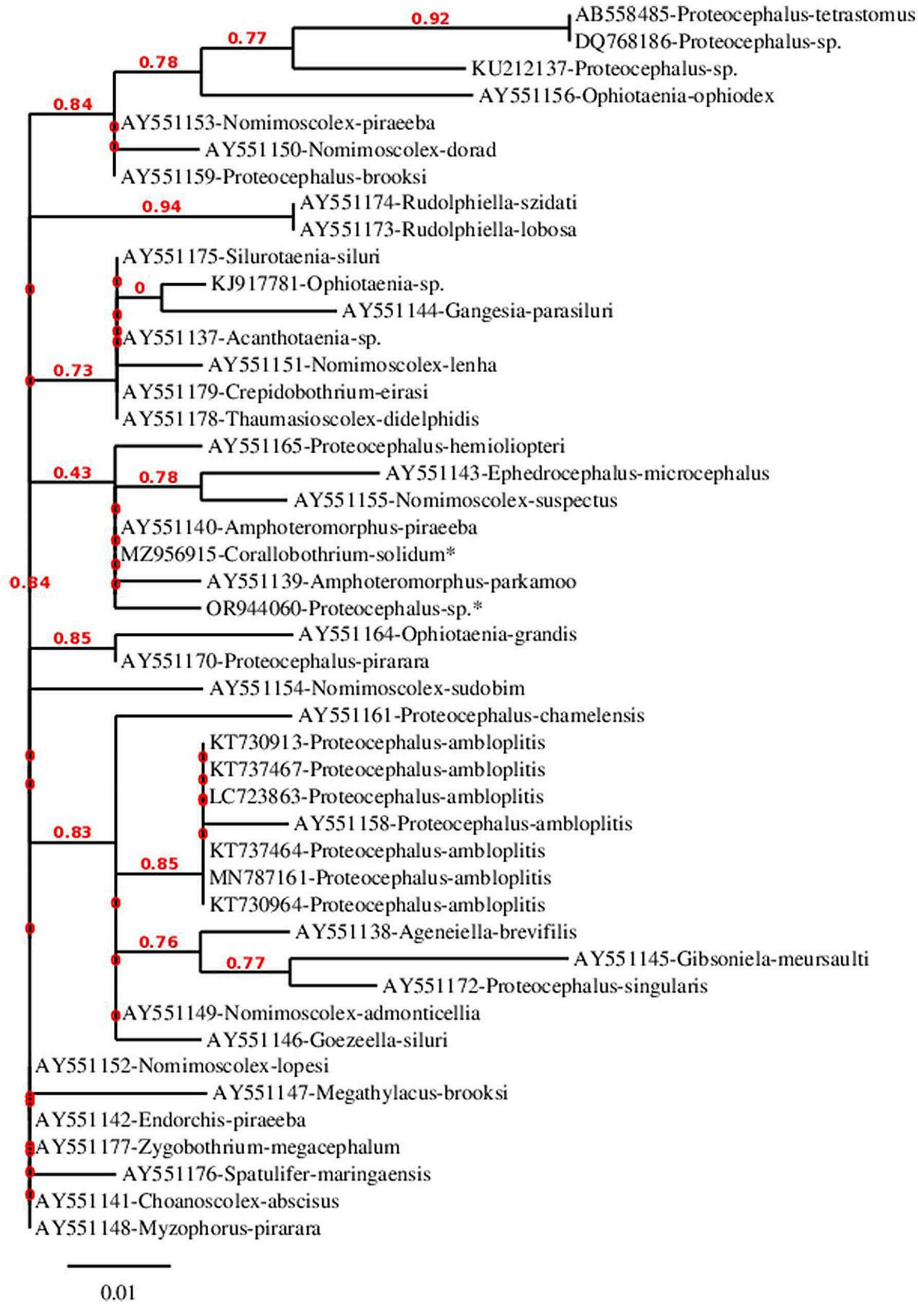
A phylogenetic tree was constructed using the Phylogeny.fr program (http://www.phylogeny.fr/) utilizing the most related and aligned complete ITS sequences of *Corallobothrium solidum* and *Proteocephalus* sp., including those identified in this study (*).The branches displayed the Bootstrap support values. The compared sequences were denoted by accession numbers, and names

Following morphological identification, *C. solidum* and *Proteocephalus* sp. identities were confirmed using specific primers. A part of the nuclear ribosomal RNA was amplified (the complete ITS region). The PCR products of both worms were ampilicons that were almost 1300 bp in length. For validation, the final sequences that were obtained were submitted to GenBank with the accession numbers MZ956915 and OR944060. They stand for the partial sequence (ITS1), complete sequence (5.8 S), and partial sequence (ITS2) for *C. solidum* and *Proteocephalus* sp., respectively. Both of the newly obtained sequences were unique from every other sequence in the GenBank databases, according to blast analysis. It is interesting to note that both sequences had a high similarity (95.77%) despite having median sequence coverage of 59%. The submitted sequence (MZ956915) of the ITS region is the first ITS sequence submission to the GenBank for the genus Corallobothrium. Only eight more sequences, ranging from 565 to 1034 bp, are available in GenBank. They include rRNA markers (28 S and 18 S) and COI but not a gene of the complete ITS region. The current limited phylogenetic tree lacks complete ITS sequences for additional potential sister taxa (the Corallobothriinae family) to *Corallobothrium* in the GenBank database, with the exception of one *Megathylacus* sequence that was found in a separate clade. Therefore, comparing the current sequence to other sequences belonging to the same genus or subfamily was not possible. The current ITS sequence of *C. solidum*, however, exhibited similarities to other ITS sequences of several members belonging to the order Proteocephalidea. On the other hand, the present ITS sequence of *Proteocephalus* sp. showed similarities to other sequences of other Proteocephalids. For example, it has similarities (80.59%, 93.39%, and 96.17%) to other ITS sequences (AB558485, MN787161, and AY551170) of *P. ambloplitis, P. tetrastomus*, and *P. pirarara* respectively. The most relevant 46 nucleotide sequences, including the present two, were used in the present limited phylogenetic analyses, Fig. (8). It appears that the two present sequences emerged in the same clade as the sequences of *Amphoteromorphus parkamoo* (acc. no. AY551139) and *A*. *piraeeba* (acc. no. AY551140). The constructed phylogram revealed that the worms examined in this study showed a very close phylogenetic relationship, with branches mostly mixed with most species. They were found to be embedded within the order Procephalidea, with considerable divergence, Fig. (8).

### Histopathological examination

**Fig. 9:**
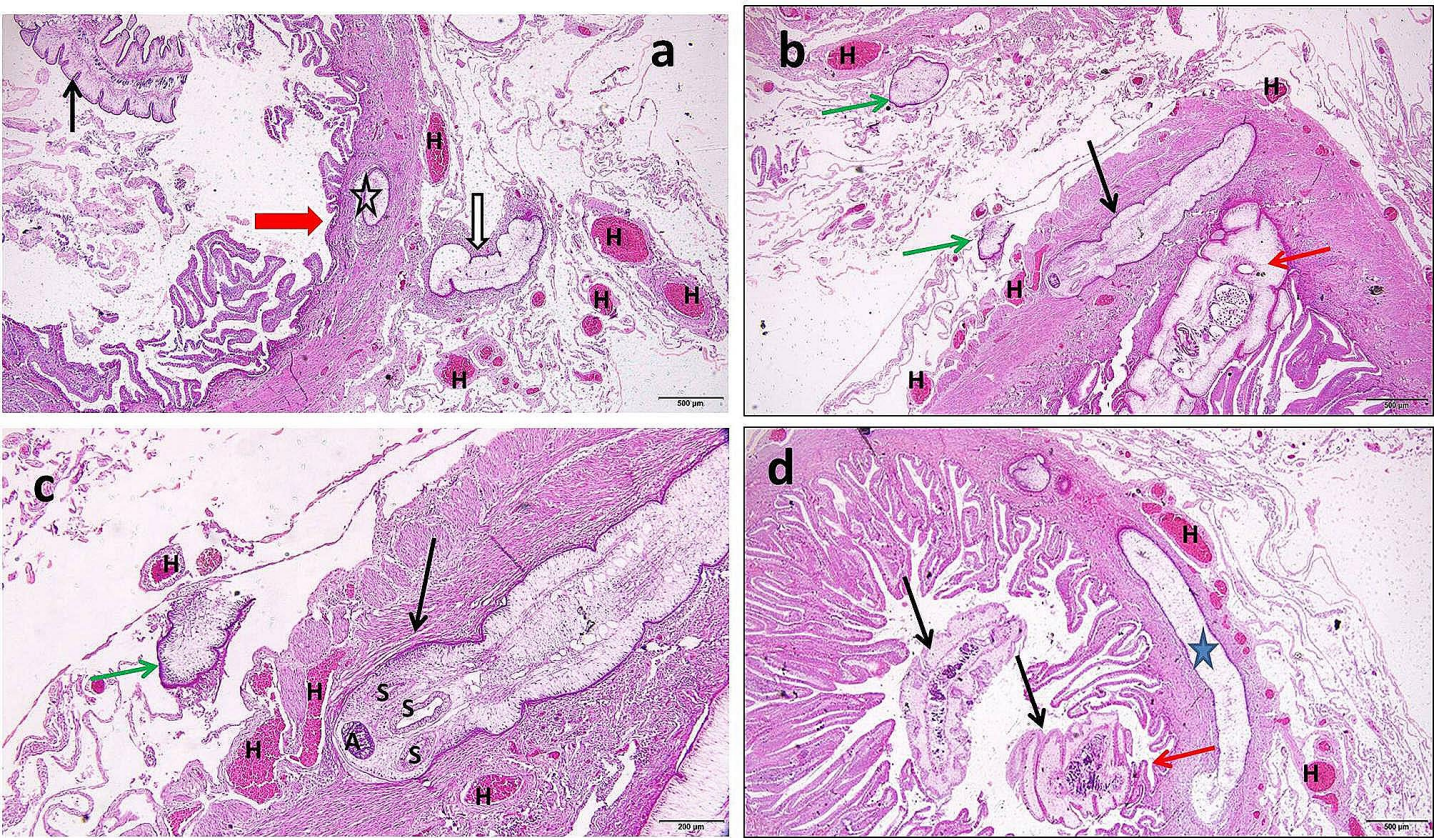
The intestine of *Malapterurus electricus*. **a**: *Corallobothrium solidum* inside the intestinal lumen (black arrow), within the intestinal wall (star) and enclosed in a sheath outside intestinal wall (black bordered white arrow). Erosions in the intestinal epithelium (red arrow) and congestion (H letters), H&E, bar = 500 μm. **b**: *Corallobothrium solidum* inside the intestinal lumen (red arrow), within the intestinal wall (black arrow) and enclosed in a sheath outside intestinal wall (green arrows), congestion (H letters), H&E, bar = 500 μm. **c**: *Corallobothrium solidum* within the intestinal wall (black arrow) and enclosed in a sheath outside intestinal wall (green arrow). Hemorrhages (H letters), H&E, bar = 200 μm. S: sucker. **d**: *Corallobothrium solidum* inside the intestinal lumen (black arrows), within the intestinal wall (star), the cestode attaching the intestinal epithelium (red arrow), congestion (H letters), H&E, bar = 500 μm

**Fig. 10:**
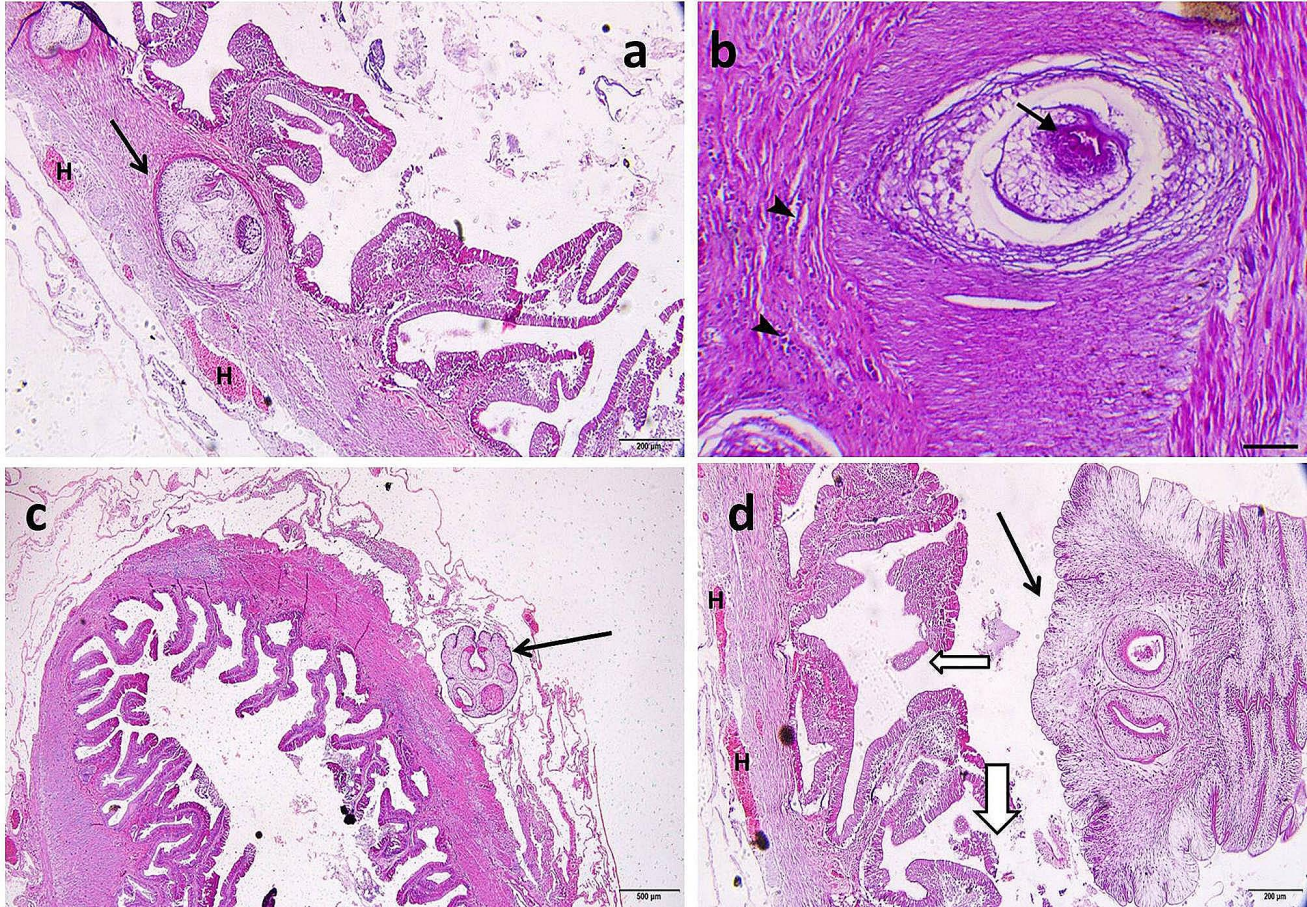
*Malapterurus electricus*^,^s intestine. **a**: Scolex of *Corallobothrium solidum* within the intestinal wall (black arrow) showing 3 suckers, congestion (H letters), H&E, bar = 200 μm. **b**: Intestinal section of *Corallobothrium solidum* showing encysted cestode parasite with apparent apical sucker (arrow) encircled with fibrous layer and mild inflammatory cells infiltration (arrowheads), H&E, X40, bar = 400 μm. **c**: Scolex of *Corallobothrium solidum* within the intestinal serosa outside the intestine showing 4 suckers (black arrow), H&E, bar = 500 μm. **d**: Scolex of *Corallobothrium solidum* in the intestinal lumen (black arrow) showing 2 suckers and erosions of the intestinal epithelium at site of attachment (black bordered white arrows), H&E, bar = 200 μm. S: suckers

**Fig. 11:**
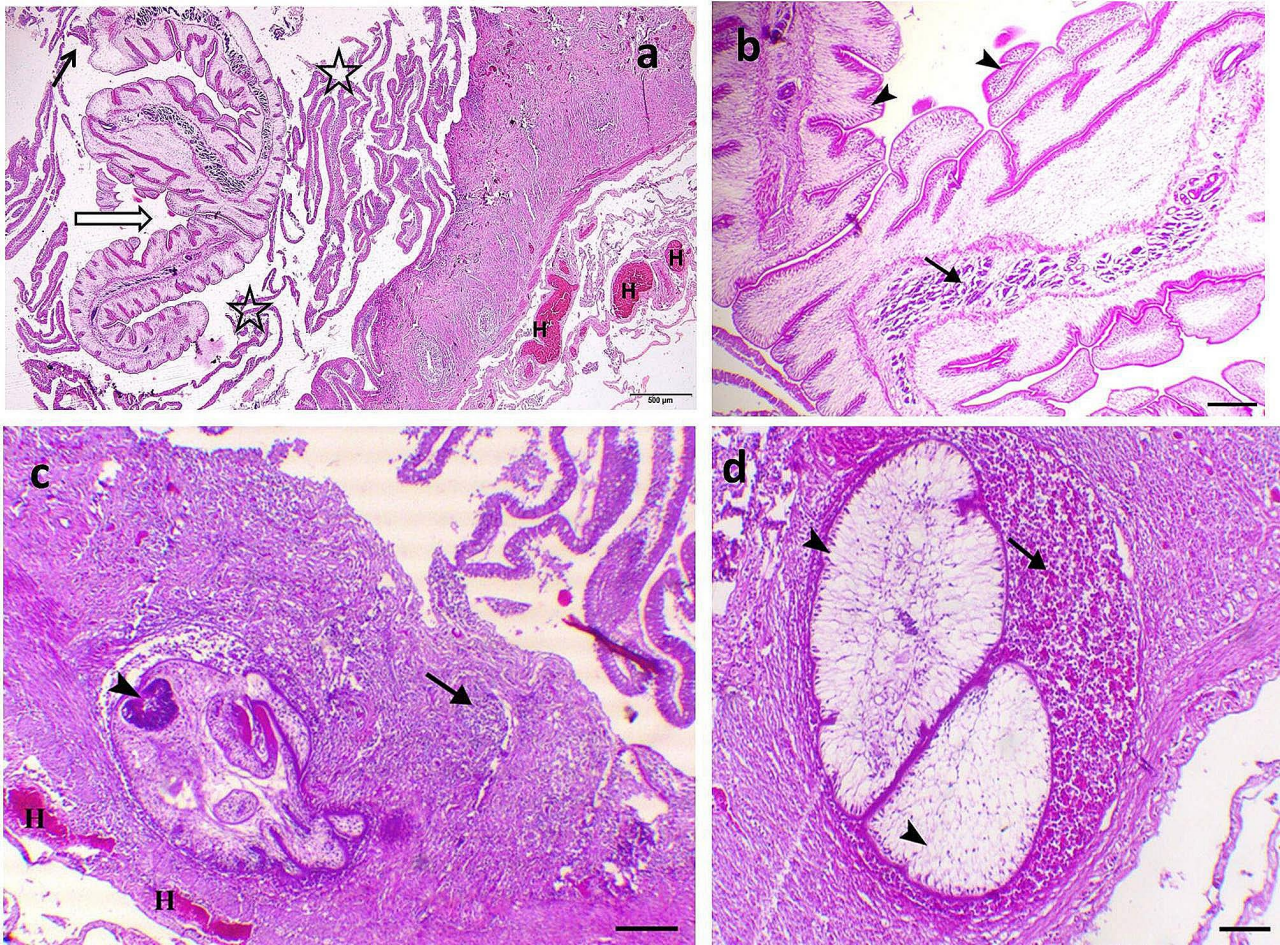
*Malapterurus electricus*^,^s intestine. **a**: Intestinal plug fromed by large *Corallobothrium solidum* of three fold-like structures (black bordered white arrow), the cestode engluf the epithelial lining of the intestine (black arrow), errosions of intestinal epithelium (stars), hemorrhages (H letters), H&E, bar = 500 μm. **b**: Section of *Corallobothrium solidum* showing leaf-like cestode of multi-villous cuticle (arrowheads) with inner gonadal tissues (arrow), H&E, X40, bar = 200 μm. **c**: Encysted *Corallobothrium solidum* with three apparent sukers with apical basophilic suckers (arrowheads) and associated with necrosis and distortion of the muscle tissues, congestion (H letter) and marked inflammatory cells infiltration (arrow), H&E, X40, bar = 400 μm. **d**: Encysted *Corallobothrium solidum* (arrowheads) associated with necrosis and distortion of the muscle tissues, hemorrhages and marked inflammatory cells infiltration including eosinophilic granular cells (arrow), H&E, X40, bar = 400 μm

The deleterious effects of *C. solidum* and *Proteocephalus* sp. on the intestines of infected fish species have been documented. The intestine of *M. electricus* infected with *C. solidum* revealed the presence of *C. solidum* attached to the intestinal wall and the larval stages attempted to penetrate the intestinal wall and sometimes they were encircled within fibrous layers infiltrated with inflammatory cells and congestion within the intestinal tissues was evident, in addition to, some larval stages were enclosed by the serosal membrane outside the intestine, Fig. (9) a, b, c, d, Fig. (10) a, b, c, d, Fig. (11) a, c, d. The infected intestine revealed necrosis and distortion of the tunica muscularis, hemorrhages, and marked inflammatory cell infiltrations, including eosinophilic granular cells within the intestinal wall, Fig. (9) a, b, c, d, Fig. (10) a, b, & Fig. (11) c, d, as well as sloughing the epithelial lining of the infected intestine at sites of cestodes attachments, Fig. (9) a, d, Fig. (10) d, & Fig. (11) (a) Section of *C. solidum* appeared as leaf-like cestode of multi-villous cuticle with inner gonadal tissues, Fig. (11) (b) Some larval stages of *C. solidum* encysted in the intestinal wall are encircled with a fibrous layer and inflammatory cell infiltration, Fig. (10) b, & Fig. (11) c, d. *Proteocephalus* sp. attached to the intestinal wall of *H. bidorsalis* may block the intestinal lumen, Fig. (7) b.

## Discussion

Numerous studies on parasitic diseases in fish in Lake Nasser have been carried out, but little is recorded on *M. electricus* and *H. bidorsalis* [18, 37, 38].

The cestodes of the genus Proteocephalus are distributed worldwide infecting a wide range of hosts, including fish, and due to the wide variation in morphological characteristics in *Proteocephalus* spp. we integrated ITS sequence and phylogenetic analysis into the observed morphological criteria of C. *solidum* and *Proteocephalus* in this study [39–41].

In addition to the adult C. *solidum* in the mid portion the intestinal tract of *M. electricus*, larval *C. solidum* (plerocercoid) were found in the coelomic cavity and underneath the skin, suggesting that *M. electricus* serves as both final and intermediate host for the same proteocephalid. This characteristic is differed from those reported by other researchers who recorded C. *solidum* only from the intestine of infected *M. electricus* [29, 30, 42]. *Proteocephalus* sp. was isolated exclusively from the mid portion of the intestinal tract of *H. bidorsalis*.

There was clear difference in the worms^,^ tegument thickness between *C. solidum* and *Proteocephalus* sp. with *Proteocephalus* sp. tegument approximately ten times thicker than *C. solidum*. Such obvious variation in the thickness may be due to variation in the pH and nature of digestive enzymes in both fish species. It is well documented that proteocephalid cestodes are protected from the intestinal proteolytic enzymes by producing proteinase inhibitors. For example, *Eubothrium rugosum* secretes proteinase inhibitor that inactivates the digestive enzymes of its host burbot (*Lota lota*) [43]. Similar finding were reported by Izvekova et al. [44] on several mechanisms performed by cestodes to protect its tegument from the powerful digestive enzymes. Whether or not the increased thickness of the *Proteocephalus* sp. found in the African giant catfish is a protective mechanism to the prevailing condition in the host intestine remains to be studied.

In this study, the suckers of *C. solidum* and *Proteocephalus* sp. cling firmly to the intestinal mucosa inducing increased mucus secretion and congestion around the site of attachment beside the larval stages of *C. solidum* even penetrate the intestinal wall, resulting in inflamed and congested intestine [6]. Increased mucus secretion in the intestine of infected fish as a defense mechanism of the fish host immune system is consistent with Hamouda [6], Bosi et al. [45] and Bosi et al. [46] who declared that intestinal helminths influence intestinal mucus secretion.

Considering this firm attachment of multiple large-size cestodes, the intestinal function of the host is definitely compromised. The harm is further amplified in some individual fish whose intestine were completely occluded affecting the food passage, reducing absorption, destructing intestinal wall and leading to the emaciation that appeared on some individual fish [39, 47]. In the same context, the cestodes have no digestive tract and took their required nutrients through the absorption of host food by their outer surface microtriches [48, 49].

The recovered cestodes were large in size and could be seen easily by naked eyes, making consumers reject the infected fish in spite of the fact that the recovered *C. solidum* and *Proteocephalus* sp. did not have zoonotic potential, resulting in economic losses.

Fortunately, the edible musculature of the infected fish was free from cestodes even in heavily infected fish.

Ibraheem [29] and Osman et al. [42] studied *M. electricus* in Egypt and discovered two species of cestodes that were highly specific to it: *C. solidum* and *Electrotaenia malepteruri*, but in this study, *C. solidum* was the only cestode recovered from this fish species in the lake. This could be due to the variety of study regions, as well as the environmental abiotic and biotic variables encountered during these studies. However, *C. solidum* plerocercoid was never reported outside the gastrointestinal tract before and these previous studies lacked molecular confirmation.

The collected *C. solidum* and *Proteocephalus* sp. of this study were morphologically similar to those recorded by Rego [9], Ibraheem [29] and Scholz et al. [30]. These studies [9, 29, 30] lacked epidemiological data about the prevalence of *C. solidum* and *Proteocephalus* sp. in *M. electricus* and *H. bidorsalis* collected from the Nile downstream from Lake Nasser, however in our study *C. solidum* and *Proteocephalus* sp. were present year-round indicating the abundance of the intermediate hosts such as cyclopid copepods, planktonic crustaceans, freshwater shrimps, and many others that harbor metacestodes [11, 12].

The females showed a higher prevalence of infection than males, and this could be a result of the random samples of collected fish, and the female fish may be more active in feeding than males, especially during spawning, to meet higher energy resources [17, 50].

The higher prevalence of infection in summer and spring seasons may be a result of suitable optimal water temperatures during these seasons, which favor the multiplication of intermediate hosts and increase fish feeding activities that facilitate parasitic transmission [42].

To our knowledge, this study is the first report of *C. solidum* and *Proteocephalus* sp. from *M. electricus* and *H. bidorsalis*, respectively, in Lake Nasser and constitutes a new geographic locale. It seems that the infection of *M. electricus* with *C. solidum* was not present in the lake, at least till 1983 [37] however, in 1984 Saoud and Wannas [38] examined 50 *M. electricus* and recorded 96% cestode infection yet these cestodes were never identified. In addition to, there is no data on *H. bidorsalis* cestode infections from Lake Nasser. Therefore there are no data to use for comparison.

In this study, *C. solidum* was recorded from *M. electricus* with a prevalence of 75% and infection intensities ranging from 1 to 50 cestodes per fish, which was different from that recorded by Ibraheem [29], who recorded it from the same fish in El-Minia Province, Egypt, with a prevalence of 50% and infection intensities ranging from 1 to 7 cestodes per fish. The difference in prevalence and intensities may be related to the changes in environmental abiotic and biotic conditions during these studies.

Molecular biology techniques such as polymerase chain reaction (PCR) and sequencing of several molecular markers are powerful tools for identifying cestode parasites and confirming their taxonomic and systematic status, as well as generic phylogenetic relationships [18, 51].

The current molecular study revealed that *C. solidum* and *Proteocephalus* sp. ITS sequences were unique yet shares some similarities to other Proteocephalidean sequences in the GenBank database. The phylogenetic analyses performed in this work allocated the most studied cestodes, which appeared scattered, to hybridized branches, with the exception of *Megathylacus*’ sequence, which is currently in a different clade. The lack of complete ITS sequences for cestodes in the GenBank database may reflect the absence of other potential sister taxa within the Corallobothriinae family to *Corallobothrium* in the phylogenetic tree of this study. Interestingly, previous phylogenetic analyses of partial sequences of the 28 S rRNA gene showed that *Corallobothrium* was not monophyletic [30]. Furthermore, according to molecular data, the subfamily with *Megathylacus* can be polyphyletic [52, 53]. The sequences of *Amphoteromorphus parkamoo* (acc. no. AY551139) and *A. piraeeba* (acc. no. AY551140), which are members of Proteocephalidea: Zygobothriinae [52], and the two current sequences all emerged in the same clade. Given the relative lengths of the branches in the phylogenetic diagram and the nodal support between them, it is probable that the order (Proteocephalidea) contains significant genetic variety. This indicates that molecular diagnosis and phylogenetic relationships are challenging, most likely because of the presence of cryptic species. These findings were consistent with the findings of Scholz et al. [30], De Chambrier et al. [51], Zygobothriinae [52], Hypsa et al. [53], Zehnder & Mariaux [54] and Waeschenbach & Littlewood [55].

The detected tapeworms had serious pathological effects on their hosts, especially the intestine, which showed degenerative changes in the intestinal mucosa with desquamation of the intestinal epithelium and enteritis. This was consistent with Hamouda [6], who recorded the same similar lesions from *Clarias gariepinus* infected with different cestodes *Polyonchobothrium clarias*, and Monobothrioides. The sloughing of the epithelial lining of the infected intestine at sites of cestodes attachment may be a result of the suckers’ attachments to the intestinal epithelium [29, 47].

*M. electricus* infected with *C. solidum* in the intestinal lumen, tunica muscularis, and enclosed by the serosal membrane covered the intestine. The infected intestine showed necrosis and distortion of the tunica muscularis, hemorrhages, and marked inflammatory cell infiltrations, including esinophilic granular cells within the intestinal wall, in which the larval stages of *C. solidum* penetrates more deeply, reaching the muscle layer and inducing destruction of the intestinal architecture due to intense inflammatory response. This was different from that recorded by Ibraheem [29], who recorded it only from the intestinal lumen, and in his histopathological study, he did not record any attack of *C. solidum* via the intestinal wall. The severity of the pathological lesions reported in *M. electricus* was much greater than that seen in *H. bidorsalis*, and this could be due to the fact that *Proteocephalus* sp. of *H. bidorsalis* did not invade the intestinal wall later and did not attach firmly to the intestinal wall.

This paper reported severe, drastic intestinal lesions associated with the two species of proteocephalid cestodes (*C. solidum* and *Proteocephalus* sp.) within the intestinal tract of the two catfish, especially *M. electricus*. Our results of severe and extensive intestinal lesions starting from the mucosa, submucosa, and tunica muscularis and ending with serosa suggest that these intestinal cestodiasis may be accompanied by disturbances in digestion and/or absorption, competition for nutrients with the host, and physiological stressor to the host due to enteritis, leading to great economic losses.

## Conclusions

This study is the first report of *C. solidum* and *Proteocephalus* sp. from *M. electricus* and *H. bidorsalis*, respectively, in Lake Nasser and constitutes a new geographic locale. They are first identified both morphologically and molecularly. The detected *C. solidum* and *Proteocephalus* sp. have serious pathological effects on their hosts, especially the intestine. Adult *C. solidum* was found in the intestine of *M. electricus* but its plerocercoid were found in the coelomic cavity and underneath the skin, suggesting that *M. electricus* serves as both final and intermediate host for the same proteocephalid. *Proteocephalus* sp. infect *H. bidorsalis* was exclusively found in the intestine. The edible musculature of the infected *M. electricus* and *H. bidorsalis* was free from cestodes even in heavily infected fish. Prevent the transfer of cestode-infected fish to a new region before treatment. Current molecular and phylogenetic analyses confirm that proteocephalid taxonomy and systematics pose ongoing issues for taxonomic classification, requiring further molecular and phylogenetic analyses for the separation of closely related genera and species as well as generic phylogenetic relationships.

## Data Availability

The data sets used in the present study are accessible on reasonable request from the corresponding author.

## Declarations

### Guidelines

All methods were carried out in accordance with relevant guidelines and regulations.

### Arrive guidelines

The authors confirm that the study was carried out in compliance with the ARRIVE guidelines.
